# Influence of certified perioperative nurses on the establishment of preoperative outpatient clinic and rate of preoperative assessment in Japan

**DOI:** 10.1038/s41598-023-51043-x

**Published:** 2024-01-12

**Authors:** Kentaro Hara, Chie Yamamoto, Shigeko Mills, Kengo Osaki, Kaoru Tokuyama, Takahiro Inoue

**Affiliations:** 1 Health Care Fee Working Committee of the Japan Perioperative Nursing Academy, Hongo Sampo Bldg. 4F Hongo 3-19-7, Bunkyo-Ku, Tokyo, 113-0033 Japan; 2 Chiba University Hospital, Chiba, Japan; 3 Department of Operation Center and Department of Nursing, National Hospital Organization Nagasaki Medical Center, Nagasaki, Japan; 4 Nagasaki University Graduate School of Biomedical Sciences, Nagasaki, Japan; 5 Ohara Healthcare Foundation Kurashiki Central Hospital, Okayama, Japan; 6 Nagano University of Health and Medicine, Nagano, Japan; 7 Kochi Medical School Hospital, Kochi, Japan; 8 The University of Tokyo Hospital, Tokyo, Japan

**Keywords:** Health care, Medical research

## Abstract

This study aimed to investigate the influence of certified perioperative nurses on preoperative outpatient clinic and preoperative assessments. The study was conducted from February 2021 to September 2022; data were collected and analyzed using a questionnaire at 247 hospitals in Japan. To analyze the factors affecting the rate of preoperative assessment with the primary endpoint of preoperative outpatient care and preoperative nursing visits, we performed multiple linear regression analysis of facility characteristics, perioperative nurse background, and the presence or absence of certified perioperative nurses. Regarding the presence or absence of a preoperative outpatient clinic, patients from 68 (52.3%) and 41 (35.0%) institutions in the enrolled and nonenrolled groups. Respectively, underwent a preoperative assessment; the rate of preoperative assessments in the enrolled group was significantly higher than that in the nonenrolled group. Multivariate analysis of factors influencing the preoperative assessment rate revealed a significant association with certified perioperative nurse attendance. Facilities with certified perioperative nurses have a significantly higher prevalence of preoperative outpatient clinic and significantly higher rates of preoperative assessments than facilities without certified perioperative nurses. Enrollment of certified perioperative nurses may lead to the improvement of the quality of preoperative nursing interventions.

## Introduction

The certified nurse specialist system aims to expand and improve the quality of nursing care by utilizing advanced nursing skills and knowledge in specific nursing fields and providing advanced nursing practice to individuals in need of care in all settings. A certified nurse has proficient skills and knowledge in a particular field of nursing and is certified by the Japanese Nursing Association^1^. A certified perioperative nurse (CPN) has passed the certification examination after completing > 800 h of the specialized perioperative curriculum at a designated educational institution in Japan. CPNs practice safety management to minimize physical and mental surgical stress, prevent secondary complications, and provide continuous nursing care in the perioperative period^1^. They practice high-level nursing care for individuals, families, and groups based on high clinical reasoning ability and disease state judgment using nursing skills and knowledge and provide guidance and consultation to nursing professionals through nursing practice.

Patients scheduled for elective surgery may experience various anxieties. In addition, patients may feel anxious and tense about the disease and its associated treatment^2^. This is because some patients find it difficult to understand and imagine a surgical procedure through explanations alone. It is crucial for patients to understand the changes that occur in their own body after surgery and receive explanations of the process from the preoperative period to postoperative discharge from the hospital. Through this, patients understand the necessity and methods of breathing techniques, pain control, and getting out of bed early and can take necessary actions to help in the recovery process. This is significantly critical as a prenursing intervention^3^. There exist two distinct categories delineating the perioperative nurse's engagement with preoperative patients: "preoperative outpatient care," initiating nursing intervention upon the patient's determination to pursue either outpatient or inpatient major surgical procedures, and "preoperative nursing visits," commencing nursing intervention upon the patient's hospital admission for the surgical endeavor^4,5^. Preoperative outpatient care is when a patient awaiting surgery goes to a hospital and is examined by a perioperative nurse before admission. Preoperative outpatient care is performed in the preoperative outpatient clinic. Nevertheless, not all hospitals in Japan possess preoperative outpatient clinics^6^. Preoperative nursing visits are conducted in hospitals devoid of a dedicated preoperative outpatient clinic. Preoperative nursing visits are visits by perioperative nurses to a patient in a hospital waiting for surgery. In preoperative outpatient care and preoperative nursing visits, perioperative nurses obtain physical characteristics data, such as height and weight, and patient information, such as medical history, allergies, and medications required for surgery.

The process, from entering the operating room to the end of surgery, and the state of the operating room are explained to the patients in advance^7^. Frequent causes for the sudden cessation of surgical interventions encompass "anxiety or skepticism," "acute illness," "changes in treatment plans," and "consent authorization "^8–10^. Perioperative nurses should ensure that patients undergoing surgery receive preoperative outpatient care and preoperative nursing visits. However, in Japan, preoperative intervention by perioperative nurses is not currently available for all patients undergoing surgery^11^. It has been reported that the rate of preoperative assessment by perioperative nurses in Japan is about 75%, and in cases where no preoperative assessment has been performed by perioperative nurses, the current situation is that nursing interventions are conducted based only on the information obtained by outpatient nurses and ward nurses^11^. CPNs often work in hospitals, and some roles include encouraging patients undergoing surgery to have a preoperative outpatient care or preoperative nursing visits and increasing the rate at which perioperative nurses perform preoperative assessments^1^. However, to date, there have been no reports on the effects of CPN enrollment on prevalence of preoperative outpatient clinic and preoperative assessments. That is, facilities with CPNs may have higher rates of preoperative assessment, whereas those without CPNs may have lower rates. This is because the CPN has established, instructed, and managed systems for facilities and perioperative nurses to improve their preoperative assessment rates^1^. Therefore, this study aimed to analyze the relationship between the presence or absence of CPN in the operating room and prevalence of preoperative outpatient clinic and the preoperative assessment rate. Furthermore, we elected to undertake a comparative analysis, employing the presence of CPN as the dependent variable. Concurrently, we considered variables such as hospital and operating room dimensions, staff qualifications, the existence of preoperative outpatient consultations, preoperative nursing consultations, the rate of preoperative evaluation implementation over a one-year span, and the incidence and rate of postoperative nursing consultations over the same duration, positing that such an approach would elucidate causal relationships. We hypothesized that the presence of CPNs is associated with an increase in the preoperative assessment rate and number of preoperative outpatient clinic. The expected influence of this study was that we will find CPNs to work effectively during the preoperative nursing visits and preoperative outpatient care and improve the quality of preoperative nursing interventions.

### Operational definitions

In this study, a preoperative assessment was defined as preoperative outpatient care and preoperative nursing visits by the perioperative nurses to patients undergoing surgery.

## Results

### Participant demographics

**Figure 1:**
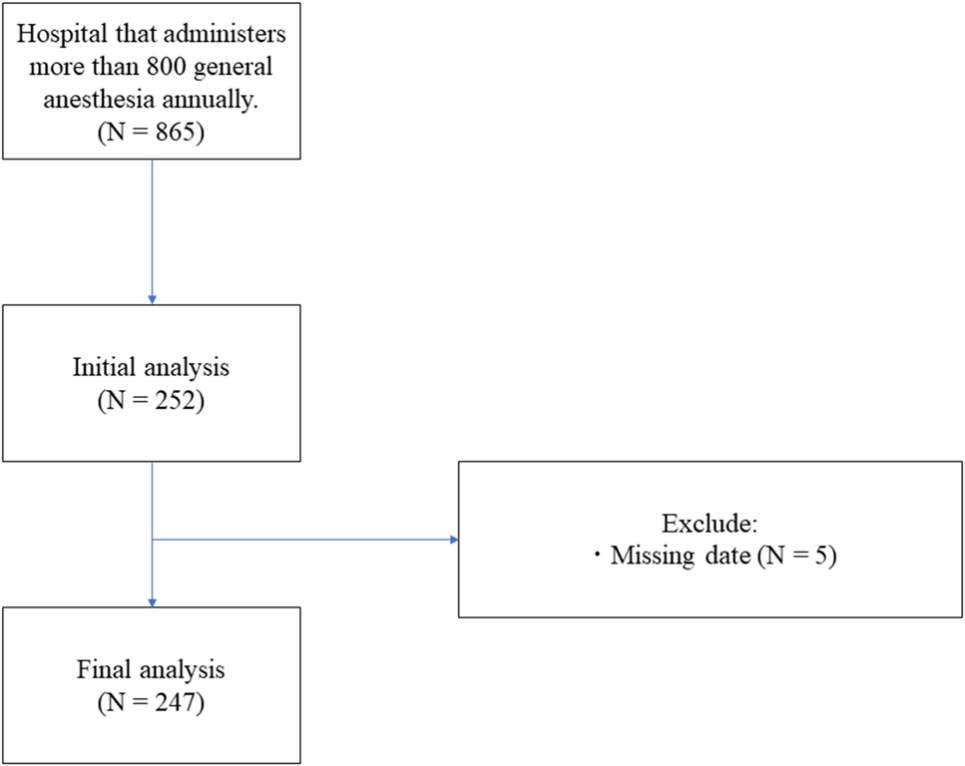
Trial STROBE diagram. Of the 865 institutions included in this study, 252 are shown to have responded. After an initial analysis, 5 hospitals with missing data are excluded, and 247 hospitals are finally analyzed.

Of the 865 institutions included in this study, 252 responded (response rate, 29.1%). After an initial analysis, 5 hospitals with missing data were excluded, and data from 247 hospitals were finally analyzed (Fig. 1). The enrolled and nonenrolled groups had 130 (52.6%) and 117 (47.4%) CPNs, respectively.

### Facility characteristics

The characteristics of the study facilities are shown in Table 1. Significant differences were found between the two groups regarding the number of intensive care units (ICU) (*p* < 0.01), advanced treatment hospitals (*p* < 0.01), licensed beds (*p* < 0.01), total operating rooms (*p* < 0.01), operations per year (*p* < 0.01), and procedures involving general anesthesia (*p* < 0.01).

**Table 1:** Facility characteristics (n = 247).

Factors	Enrolled group (n = 130)	Nonenrolled group (n = 117)	*p*-value
Hospital location			0.58
Ordinance-designated city	45 (34.6%)	36 (30.8%)	
Nonordinance-designated city	85 (65.4%)	81 (69.2%)	
Intensive care unit			< 0.01*
Yes	116 (89.2%)	66 (56.4%)	
No	14 (10.8%)	51 (43.6%	
Advanced treatment hospital			< 0.01*
Yes	58 (44.6%)	22 (18.8%)	
No	72 (55.4%)	95 (81.2%)	
Number of hospital beds			< 0.01*
< 200	4 (3.1%)	19 (16.2%)	
200–399	29 (22.3%)	51 (43.6%)	
400–599	41 (31.5%)	36 (30.8%)	
> 600	56 (43.1%)	11 (9.4%)	
Number of operating rooms	10 (3–26)	6 (1–23)	< 0.01*
Number of surgeries annually	4632 (1327–12,742)	2592 (200–9662)	< 0.01*
Number of general anesthesia cases annually	2913 (856–8800)	1603 (130–6361)	< 0.01*

Values are presented as medians (interquartile ranges) or numbers (percentages). The Mann–Whitney U test was used for the comparison of ordinal data. Nominal data were compared using the chi-squared or Fisher’s exact test.

*Significant difference between groups.

### Perioperative nurse background

The perioperative nurse background of the study facilities is shown in Table 2. The enrolled group had significantly more perioperative nurses than the nonenrolled group (*p* < 0.01); however, no significant difference was noted in the number of perioperative nurses per operating room between the two groups (*p* = 0.13). Of the 130 facilities, 73.2% had 1 CPN.

**Table 2:** Perioperative nurse characteristics (n = 247).

Factors	Enrolled group (n = 130)	Nonenrolled group (n = 117)	*p*-value
Number of perioperative nurses	34 (10–97)	21 (4–72)	< 0.01**
Number of perioperative nurses per operating room	3.6 (1.9–6.8)	3.3 (1.8–5.7)	0.13
< 3.0	22 (16.9%)	25 (21.6%)	
3.0–3.4	20 (15.4%)	33 (28.5%)	
3.5–3.9	40 (30.8%)	27 (23.3%)	
4.0–4.4	24 (18.5%)	17 (14.7%)	
> 4.5	24 (18.5%)	14 (12.1%)	
Certified perioperative nurses (n)		–	–
One	95 (73.2%)		
Two	31 (23.8%)		
Three	2 (1.5%)		
Four	2 (1.5%)		

Values are presented as medians (interquartile ranges) or numbers (percentages). The Mann–Whitney U test was used for the comparison of ordinal data.

*Significant difference between groups.

### Preoperative assessment and preoperative and postoperative nursing visits

The preoperative and postoperative relationships are shown in Table 3. Regarding the presence or absence of a preoperative outpatient clinic, patients from 68 (52.3%) and 41 (35.0%) institutions in the enrolled and nonenrolled groups. Respectively, underwent a preoperative assessment; the rate of preoperative assessments in the enrolled group was significantly higher than that in the nonenrolled group (*p* = 0.01). Regarding the preoperative assessment rate, the medians (quartile ranges) of the enrolled and nonenrolled groups were 90.0% (70.0–99.0%) and 75.0% (54.5–95.0%), respectively, indicating a significantly higher preoperative assessment rate in the enrolled group than in the nonenrolled group (*p* = 0.01). Regarding conducting postoperative nursing visits, the enrolled group was significantly more likely to conduct postoperative nursing visits than the nonenrolled group (*p* = 0.03). No significant difference was observed in the rate of postoperative nursing visits between the two groups.

**Table 3:** Preoperative assessment and preoperative and postoperative visits (n = 247).

Factors	Enrolled group (n = 130)	Nonenrolled group (n = 117)	*p*-value
Preoperative outpatient care/ nursing visits			0.01*
Preoperative outpatient care only	8 (6.2%)	4 (3.4%)	
Preoperative nursing visits only	62 (47.7%)	73 (62.4%)	
Both conducted	60 (46.1%)	37 (31.6%)	
Neither conducted	0 (0.0%)	3 (2.6%)	
Preoperative assessment rate (%)	90.0 (70.0–99.0)	75.0 (54.5–95.0)	0.01*
Postoperative nursing visits			0.03*
Yes	112 (86.2%)	88 (75.2%)	
No	18 (13.8%)	29 (24.8%)	
Postoperative nursing visits rate (%)	44.0 (11.5–80.0)	39.0 (14.3–80)	0.93

Values are presented as medians (interquartile ranges) or numbers (percentages). The Mann–Whitney U test was used for the comparison of ordinal data. Nominal data were compared using the chi-squared or Fisher’s exact test.

*Significant difference between groups.

Multiple linear regression analysis of the factors influencing the preoperative assessment rate, including facility characteristics, personal background, and the presence or absence of CPNs, revealed a significant association with CPN presence (R^2^ = 0.571, adjusted R^2^ = 0.571) (B = 6.7, *p* < 0.01) (Table 4).

**Table 4:** Analysis of factors influencing the rate of preoperative assessment (n = 247).

Factors	B	SE	t	*p*-value	95%CI
Hospital Location (ordinance-designated city)	− 4.31	1.82	− 0.95	0.32	− 1.88 to 5.58
Intensive care unit (yes)	− 2.42	2.53	− 0.98	0.32	− 7.33 to 2.45
Advanced treatment hospital (yes)	− 4.48	2.37	− 1.93	0.05	− 1.88 to 5.58
Number of operating rooms	0.81	1.93	0.38	0.44	− 3.15 to 4.66
Number of surgeries annually	− 0.93	1.65	0.22	0.94	− 0.08 to 0.02
Number of general anesthesia cases annually	0.01	0.34	0.58	0.76	− 0.02 to 0.01
Number of perioperative nurses per operating room	0.45	2.60	0.17	0.86	− 4.68 to 5.58
Certified perioperative nurse (enrolled)	6.7	2.5	2.62	< 0.01**	1.46–11.57
Preoperative outpatient clinic (yes)	4.3	2.1	2.54	0.03*	1.01–5.97

*Significant difference between groups.

## Discussion

This study aimed to analyze the relationship between the presence or absence of CPN in the operating room and prevalence of preoperative outpatient clinic and the preoperative assessment rate. The results from this study demonstrated significant differences between the enrolled and nonenrolled CPN groups in all facility characteristics, except the location. Skilled professionals, such as certified and professional nurses, have different requirements depending on the hospital size. In addition, it suggest that CPNs tend to be placed in acute care hospitals in which patients may require surgery. That is, the CPNs were placed in an environment where they could intervene preoperatively with patients undergoing surgery.

From the results of facility characteristics and medical staff backgrounds, it can be inferred that the size of facilities and staffing in the enrolled group were larger than those in the nonenrolled group. With an increase in the number of surgeries, appropriate staffing with operating room nurses is critical^12–14^. We believed that the preoperative assessment rate would be high because there were several perioperative nurses, with the number of operations increasing according to the facility characteristics of the enrolled and nonenrolled groups. However, no significant difference was found in the number of patients per operating room between the enrolled and nonenrolled groups, suggesting that there may not be any additional personnel to perform preoperative assessments. Consequently, predicated on the outcomes of the multiple regression analysis, it appears plausible that the augmented preoperative consultation rate within the registration group does not solely emanate from an ample cadre of operating room personnel for preoperative assessments. Rather, the involvement of CPNs might exert influence on this elevated preoperative assessment frequency. For patients for whom surgery is decided, starting physical and psychological preparations before surgery is crucial to provide a safe and secure surgery^15^. For patients undergoing surgery, nurse-led preoperative outpatient visits have been introduced in various specialties to prevent cancellations before surgery^16–18^. In addition, there is no difference in costs between professional nurse- and physician-led preoperative outpatients when examined using economic models based on patient satisfaction with care and completeness^19^. Therefore, setting up a preoperative outpatient clinic and intervening from an early stage may be beneficial for patients undergoing surgery and for medical professionals. The enrolled group had a significantly higher preoperative assessment rate than the nonenrolled group. An effective preoperative assessment improves patient outcomes by ensuring that patients are adequately prepared for anesthesia, surgery, and the postoperative period^20^. Moreover, it can improve hospital efficiency by shortening patient waiting time and allowing for an early discharge. Consequently, a preoperative assessment within a preoperative outpatient clinic, fortified by the presence of a CPN, may serve as an invaluable mechanism to uphold patient safety and facilitate a more structured hospital sojourn. In the operating rooms of Japanese hospitals, CPNs are not always deployed. Therefore, for the safety and security of patients undergoing surgery, hospital operating rooms should deploy CPNs and increase the rate of preoperative assessment.

This study had a few limitations. Within our study, encompassing 865 facilities, a mere 247 provided responses, a circumstance that could be delineated as a potential limitation predisposing to bias. The 247 hospitals in this study were active in preoperative evaluation and may have had a latent tendency to incorporate CPNs or an inherent motivation to foster and acquire proficiency in CPN. In addition, whilst we discerned a potential causal nexus between CPNs and the preoperative outpatient clinic and rates of preoperative assessment, elucidating the precise activities and involvements of CPNs influencing these rates remained elusive within our analysis. In the future, we aim to conduct additional studies on CPNs, including quantitative and qualitative studies on the backgrounds of CPNs and the activities they performed in the affiliated facilities.

This study suggests that CPN enrollment may contribute to the establishment of preoperative outpatient. We believe that the establishment of this preoperative outpatient clinic is closely related to an improvement in the preoperative assessment rates. Preoperative outpatients clinics are more efficient, as preoperative interviews do not require hospitalization. Therefore, we infer that the preoperative assessment rate was higher in the facilities with CPNs, which had preoperative outpatient clinics, because they could interview patients scheduled for surgery without failure. This is suggested by the results of the multivariate analysis of the preoperative assessment rates.

Facilities with CPNs have a significantly higher prevalence of preoperative outpatient clinic and significantly higher rates of preoperative assessments than facilities without CPNs. Our investigation underscores the prospective efficacy of CPNs in enhancing preoperative outpatient care. Consequently, we posit that advocating for outpatient care will bolster preoperative evaluation rates and engender a holistic patient assessment.

## Methods

### Study design and ethical considerations

The study design was a multicenter retrospective cross-sectional study. This study was approved by the Ethics Committee of the Japan Perioperative Nursing Academy (no. 14). All methods were performed in accordance with relevant guidelines and regulations. This study was conducted in accordance with the ethical standards of the Declaration of Helsinki (1964) and its amendments. Informed consent was confirmed by completing a questionnaire, and the Ethics Committee of the Japan Perioperative Nursing Academy confirmed this method of consent. All observational protocols were approved by the institutional and licensing committees of the Japan Perioperative Nursing Academy. The study design adhered to the Strengthening the Reporting of Observational studies in Epidemiology guidelines^21^. In this study, the questions were entered anonymously so that the names of the individuals and institutions to which they belonged were not identified. Consent was provided by completing the questionnaire. In addition, the system of the questionnaire tool “Questant” (https://questant.jp) used in the survey in this study was monitored 24 h a day, 365 days a year. "Questant" serves as a web-based instrument for questionnaire administration, encompassing both nominal and ratio scales.

### Study setting and population

This multicenter, retrospective cross-sectional study was conducted between February 2021 and September 2022. The study was conducted at 865 hospitals in Japan that administered general anesthesia in > 800 procedures annually to standardize the number of operations and other matters as facilities that provide comprehensive and advanced medical care, in accordance with the requirements of the additional comprehensive hospitalization system. Facilities accredited with in excess of 800 cases per annum of general anaesthesia were sourced from the database accessible on the Ministry of Health, Labour and Welfare website^22^.

### Outcome and data collection

Regarding the scale of hospitals and operating rooms, the number of hospital beds, operating rooms (all operating rooms), annual number of operations, and annual number of general anesthesia procedures were surveyed. In relation to the operating room staff, the number of perioperative nurses, number of years of experience, presence or absence of CPNs, and number of such nurses were registered. Institutions with CPNs were categorized into the enrollment group and institutions without CPNs into the nonenrolled group. We investigated the presence or absence of preoperative outpatient clinic and preoperative nursing visits, rate of preoperative assessments (1 year), presence or absence of postoperative nursing visits, and rate of implementation (1 year). The data covered the fiscal year 2020–2021. The primary endpoint was the preoperative assessment rate, including preoperative outpatient care and preoperative nursing visits. The secondary endpoints were the presence or absence of a preoperative outpatient clinic, presence or absence of a postoperative nursing visits, and rate of nursing visits. The data was collected using a questionnaire with answers input to Questant (https://questant.jp), an application that allowed input via the Internet, and a quantitative survey was conducted. The questionnaire was created independently; all researchers confirmed that the content was in line with the study objectives, and it was used after the pretest.

### Data analysis procedures

The scale of hospitals and operating rooms, in relation to the operating room staff and preoperative outpatient care, are presented as median (interquartile range). The sample size could not be calculated because there is no sample data for CPN. To analyze the factors affecting the rate of preoperative assessment by the primary endpoint of preoperative outpatient care and preoperative nursing visits, we performed multiple linear regression analysis by facility characteristics, personal backgrounds, and the presence or absence of CPNs. In addition, the CPN enrolled group was compared with the nonenrolled group using the Student’s t-test, and differences were considered significant at a significance level of 5%. Secondary endpoints were compared using the chi-squared test, Fisher's exact test, Mann‒Whitney U test, and Student’s t-test. Differences with a significance level of 5% were considered statistically significant. The statistical software JMP® 15 (SAS Institute Inc., Cary, NC, USA) was used in this study.

### Supplementary Information

Supplementary Information.

## Data Availability

The questionnaire data for this study can be found in Kentaro, Hara (2023), “Certified Perioperative Nurses in the Establishment of Preoperative Outpatient Visits and Rate of Preoperative Assessment in Japan: A Cross-sectional Study,” Mendeley Data, V1, 10.17632/vkx6m3n57h.1. The datasets used and/or analyzed during the current study are available from the corresponding author upon reasonable request.

## Abbreviation

CPN: Certified perioperative nurse
