# Supplementary material for: Influence of certified perioperative nurses on the establishment of preoperative outpatient clinic and rate of preoperative assessment in Japan
Source: Sci Rep. 2024 Jan 12;14:1192. doi: 10.1038/s41598-023-51043-x (PMC10786942; doi:10.1038/s41598-023-51043-x)
Supplement: Supplementary file 1 — Supplementary Information. [file 41598_2023_51043_MOESM1_ESM.docx]

**Online Resource 1: STROBE Statement Checklist**

|  | Item No. | Recommendation | Page  No. | Relevant text from manuscript |
| --- | --- | --- | --- | --- |
| **Title and abstract** | 1 | (*a*) Indicate the study’s design with a commonly used term in the title or the abstract | 1 | Title and Abstract |
|  |  | (*b*) Provide in the abstract an informative and balanced summary of what was done and what was found | 4–5 | Abstract |
| Introduction | | | |  |
| Background/rationale | 2 | Explain the scientific background and rationale for the investigation being reported | 6–8 | Background |
| Objectives | 3 | State specific objectives, including any prespecified hypotheses | 7–8 | Background |
| Methods | | | |  |
| Study design | 4 | Present key elements of study design early in the paper | 8 | Methods |
| Setting | 5 | Describe the setting, locations, and relevant dates, including periods of recruitment, exposure, follow-up, and data collection | 8–9 | Methods |
| Participants | 6 | Give the eligibility criteria, and the sources and methods of selection of participants. Describe methods of follow-up | 8–9 | Methods |
| Variables | 7 | Clearly define all outcomes, exposures, predictors, potential confounders, and effect modifiers. Give diagnostic criteria, if applicable | 8–9 | Methods |
| Data sources/ measurement | 8 | For each variable of interest, give sources of data and details of methods of assessment (measurement). Describe comparability of assessment methods if there is more than one group | 8–9 | Methods |
| Bias | 9 | Describe any efforts to address potential sources of bias | 8–9 | Methods |
| Study size | 10 | Explain how the study size was arrived at | 8–9 | Methods |

Continued on next page

| Quantitative variables | 11 | Explain how quantitative variables were handled in the analyses. If applicable, describe which groupings were chosen and why | 9–10 | Methods (Measurements) |
| --- | --- | --- | --- | --- |
| Statistical methods | 12 | (*a*) Describe all statistical methods, including those used to control for confounding | 9–10 | Methods (Statistical Analysis) |
|  |  | (*b*) Describe any methods used to examine subgroups and interactions |  | Not applicable |
|  |  | (*c*) Explain how missing data were addressed |  | Not applicable |
|  |  | (*d*) If applicable, explain how loss to follow-up was addressed |  | Not applicable |
|  |  | (*e*) Describe any sensitivity analyses | 9–10 | Methods (Statistical Analysis) |
| Results | | | | |
| Participants | 13 | (a) Report numbers of individuals at each stage of study—eg numbers potentially eligible, examined for eligibility, confirmed eligible, included in the study, completing follow-up, and analysed | 10–11 | Results |
|  |  | (b) Give reasons for non-participation at each stage | 10–11 | Results |
|  |  | (c) Consider use of a flow diagram | 10–11 |  |
| Descriptive data | 14 | (a) Give characteristics of study participants (eg demographic, clinical, social) and information on exposures and potential confounders | 10–11 and Table 1 | Results and Table 1 |
|  |  | (b) Indicate number of participants with missing data for each variable of interest | 10–11 | Results |
| Outcome data | 15 | Report numbers of outcome events or summary measures over time | 10–11 | Results |
| Main results | 16 | (*a*) Give unadjusted estimates and, if applicable, confounder-adjusted estimates and their precision (eg, 95% confidence interval). Make clear which confounders were adjusted for and why they were included | 10–11 | Results |
|  |  | (*b*) Report category boundaries when continuous variables were categorized | 10–11 | Results |
|  |  | (*c*) If relevant, consider translating estimates of relative risk into absolute risk for a meaningful time period | 10–11 | Results |

Continued on next page

| Other analyses | 17 | Report other analyses done—eg analyses of subgroups and interactions, and sensitivity analyses | 10–11 | Results |
| --- | --- | --- | --- | --- |
| Discussion | | | | |
| Key results | 18 | Summarise key results with reference to study objectives | 12–14 | Discussion |
| Limitations | 19 | Discuss limitations of the study, taking into account sources of potential bias or imprecision. Discuss both direction and magnitude of any potential bias | 12–14 | Discussion |
| Interpretation | 20 | Give a cautious overall interpretation of results considering objectives, limitations, multiplicity of analyses, results from similar studies, and other relevant evidence | 12–14 | Discussion |
| Generalisability | 21 | Discuss the generalisability (external validity) of the study results | 12–14 | Discussion |
| Other information | |  | | |
| Funding | 22 | Give the source of funding and the role of the funders for the present study and, if applicable, for the original study on which the present article is based | 15 | 15 |
